# Effects of Side Profile on Acoustic Streaming by Oscillating Microstructures in Channel

**DOI:** 10.3390/mi13091439

**Published:** 2022-08-31

**Authors:** Lin Lin, Haojie Dang, Rongxin Zhu, Ying Liu, Hui You

**Affiliations:** 1 Key Laboratory of Disaster Prevention and Structural Safety of Ministry of Education, Guangxi University, Nanning 530004, China; 2 School of Mechanical Engineering, Guangxi University, Nanning 530004, China; 3 Guangxi Key Lab of Manufacturing System and Advanced Manufacturing Technology, Nanning 530003, China; 4 Guangxi Key Laboratory of Disaster Prevention and Engineering Safety, Guangxi University, Nanning 530004, China

**Keywords:** acoustic streaming, perturbation theory, side profile, sidewall, upper wall

## Abstract

In microchannels, microstructure-induced acoustic streaming can be achieved at low frequencies, providing simple platforms for biomedicine and microfluidic manipulation. Nowadays, microstructures are generally fabricated by photolithography or soft photolithography. Existing studies mainly focused on the projection plane, while ignoring the side profile including microstructure’s sidewall and channel’s upper wall. Based on the perturbation theory, the article focuses on the effect of microstructure’s sidewall errors caused by machining and the viscous dissipation of upper wall on the streaming. We discovered that the side profile parameters, particularly the gap (gap *g* between the top of the structure and the upper wall of the channel), have a significant impact on the maximum velocity, mode, and effective area of the streaming.To broaden the applicability, we investigated boundary layer thickness parameters including frequency and viscosity. Under different thickness parameters, the effects of side profile parameters on the streaming are similar. But the maximum streaming velocity is proportional to the frequency squared and inversely proportional to the viscosity. Besides, the ratio factor θ of the maximum streaming velocity to the vibration velocity is affected by the side profile parameter gap *g* and sidewall profile angle α.

## 1. Introduction

Acoustic Streaming is a non-zero time-averaged flow due to the nonlinearity created by acoustic waves in fluid propagation [1]. It forms through three viscous dissipations: acoustic energy attenuation in space, acoustic wave scattering, and friction between the fluid and the wall [2]. These three types of dissipation often coexist in real fluids. Microfluidic chips and acoustic streaming can now be combined thanks to the rapid advancement of MEMS technology. Combining streaming with microfluidic chips can achieve advantages including low consumption, high efficiency, integrated platforms, good biocompatibility, easy manipulation, and contactless [3,4,5,6,7]. Thus, acoustic streaming has become an important tool for particle manipulation [8,9,10,11,12], cell capture [13], micromixing [14], micropump [15], material concentration [16], and chemical reactions [17].

**Figure 1:**
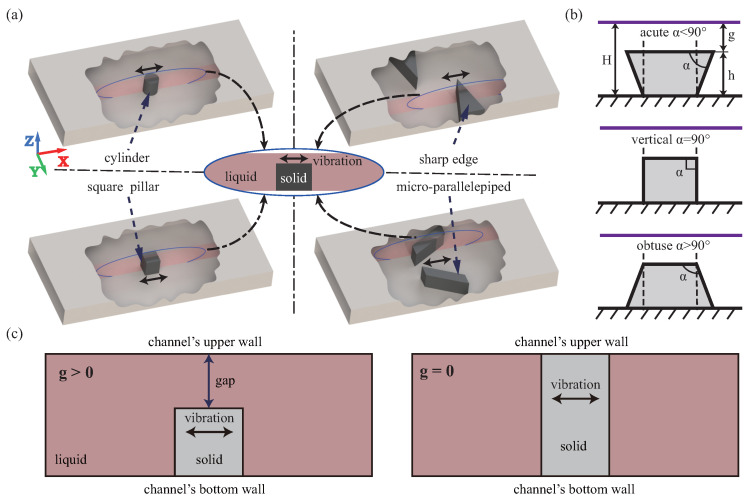
The diagram of microstructure-induced streaming device and microstructure sidewall. (**a**) The common microstructures of devices in the channel include microcylinders, sharp edges, micro square pillars, and micro parallelepipeds, where solid line with black arrow is the direction of microstructure vibration. The longitudinal section of the microstructure is enlarged and displayed in the middle, in which the pink plane is the section, and the blue ellipse is the section outline. The pink plane is one of a series of viewing planes at various focal lengths. (**b**) The ideal sidewall of microstructure, prepared by photolithography or soft lithography, is vertical as the dotted line which is the designed optical path through the masks. But the sidewall profile angle α caused by errors in actual processing can be classified into acute, vertical, and obtuse. Where *H* is the channel height, *h* is the microstructure height, *g* is the gap, and the purple line denotes the upper wall. (**c**) The upper wall’s position of microstructure induced streaming in channel can be classified as g>0 and g=0. The latter implies that the channel’s upper wall is connected to the microstructure, which is not the case in our case.

Because of the chip’s size effect, the viscous layer’s dissipation between the fluid and the wall dominates in microfluidic acoustic streaming, and the resulting acoustic streaming can be defined as the boundary layer streaming [18]. Bubbles are one of the main ways to induce streaming and have been the focus of past research [19,20]. It can generate a strong streaming, but the resonant frequency of the excitation is highly dependent on the size of the microbubble, while uniformizing the size of the microbubble achieves difficult with limited temporal stability [21]. This issue can be avoided by the microbubble-free induced streaming. To obtain sufficiently strong boundary layer streaming, standing waves must be introduced into the fluid if there is no obstruction in the channel. Due to the microchannel’s width, the excitation frequency must be higher than kHz [22,23], which undoubtedly increases manufacturing difficulty and cost and enhances requirements for the excitation circuits, such as power amplification, which must be suitable for higher frequencies. Various microstructures prepared by MEMS process are introduced into microchannels, such as microcylinders [24,25], sharp edges [26,27,28], micro square pillars [29], and micro parallelepipeds [30], as Figure 1a. With the microstructure’s participation, much viscous loss occurs in the fluid near its surface, making it possible to generate a strong boundary layer streaming at low frequencies.

Microstructure-induced flow braking of laminar flow in microchannels has received extensive attention in recent years. The fluids in microchannels have characteristic sizes ranging from tens to hundreds of microns, and their Reynolds number Re≪1, implying that viscous forces dominate [31]. Therefore, the geometric profile of the microstructure within the microchannel becomes a key factor, and many articles have been devoted to this aspect. The researchers designed different shapes for various requirements. The cylinder is applied 138 kHz elliptical vibration to produce a high-speed rotational flow that enables the rotation of polystyrene particles up to 5000 rpm [32]. The sharp edges allow cells and organisms to rotate [5], while asymmetrical sharp edges enable diatom cells to rotate up to 1800 rpm [26]. In addition, Valerie et al. designed 9 different shapes of the microstructure, and theoretical studies revealed that the shape influences the distribution, pattern, and intensity of the streaming [33]. Even the same shape, angle and tilt angle cannot be ignored. The smaller the angle of the sharp edge, the larger the vortex range formed by the streaming, and the better the mixing effect [34]. For sharp edges at larger angles, the resulting secondary near-wall vortex develops along the wedge side [35]. Moreover, the sharp edges inclined at 60° can complete the non-contact transfer of micro-agents [36]. Even the microstructure’s tip curvature is a non-negligible factor, the smaller radius of curvature of the sharp edge tip, the stronger the streaming [37]. The micron scale surface profiles in the channel can also act as microstructures to enhance streaming. When the amplitude of the micro-scale profile comparable to the viscous boundary layer, the streaming can be enhanced by up to 100-fold [38]. The current studies of acoustic streaming focus on the projection plane, which is simplified to assume that the streaming is the same for each section along the Z-axis. This is reasonable in the ideal case of infinitely high microstructures, but the height is finite. In addition, without considering the side profile will ignore the difference in streaming distribution on the Z-axis, which will affect the particle motion trajectory, cell rotation, and particle aggregation state. Hayakawa et al. apply micropillars to achieve three-dimensional rotation of particles [39], which demonstrates a non-uniform distribution of streaming along the Z-axis.

Microstructures are typically fabricated directly by photolithography or by molding technology using a photolithographic positive mold. The light projection surface can be better guaranteed, but considering the light scattering and uneven light intensity along the thickness, the sidewall profile isn’t ideal (vertical) in real [40]. In the case of negative-working photoresist, more light is absorbed near the exposure surface. The sidewall profile is inverted trapezoid when frontal exposure, while trapezoidal when back exposure. In addition, the viscous dissipation near the microchannel wall caused by oscillating microstructure is no reason to ignore. When circular oscillations are applied to a cylinder with a diameter of 200 μm and a height of 100 μm, strong upward and downward vortices appear in the vertical flow field at the cylinder’s top where the height of fluid domain is constant (200 μm) [39]. However, the study only analyzed the fixed upper wall position. We take the changing upper wall position as a factor in the side profile.

Although the non-uniform distribution of streaming caused by side profile has been widely applied, the mechanism of the effect of side profile on streaming is lacking. This article focuses on the effect of the side profile on the streaming, investigates the changes of different side profiles and their various application conditions on the streaming, and reveals the internal rules and their inducements. This will provide the theoretical basis for particle or cell localization displacement, attitude rotation, and trajectory prediction on the side. Based on perturbation theory (PT) [41], we design the 2D model to analyze the effect of side profile on the microstructures-induced streaming in the channel, including the microstructure’s sidewall profile and the upper wall’s position. To investigate the effect of the sidewall profile on the streaming, we defined the gap as the space between the channel and microstructure to evaluate the position of the upper wall, and designed three different sidewall profiles, including acute, vertical, and obtuse angles, as Figure 1b. This article focuses on the effects of manufacturing-induced sidewall profile errors (profile angle) and channel upper wall’s viscous dissipation. PT simplifies the Navier-Stokes (NS) equation by solving step by step, which reduces the computational difficulty compared with the direct method and maintains good computational accuracy under the “weak disturbances” framework [37]. In our study, the ratio ζ of the vibration velocity amplitude $V_{a}$ and the sound velocity $c_{0}$, $\zeta =V_{a}/c_{0}\ll 1$, thus PT is reasonable. We introduce quantitative parameters (maximum streaming velocity $V_{2max}$ and effective area $S_{A}$) and qualitative parameter (model) to describe the effect on streaming’s characteristics. Then, to investigate the influence of the side profile, the sidewall profile angle α and height *h* (describing the microstructure features), and the gap *g* parameter (describing the position of the upper wall) are introduced. Since the thickness of the viscous layer δ ($\delta =\sqrt{2\eta /\omega }$) cannot be ignored in boundary layer streaming. Therefore, this article also investigates the effect of side profile on streaming under different boundary layer thickness parameters (including viscosity η and frequency *f*). It should be noted that the study is aimed at the case where there is a space (g>0) between the channel’s upper wall and the microstructure, as illustrated in Figure 1c. This is due to the fact that, on the one hand, the existence of the space will cause much viscous dissipation in the gap, which will significantly affect the streaming, so the upper wall should be considered in this case; on the other hand, if g=0 (the channel’s upper wall and the structure is connected), when the transducer excites the substrate (the channel’s bottom wall) at resonance, the microstructure is driven to vibrate causing its deformation, and the deformation mode is complex and greatly affected by the material of the channel’s upper wall (such PDMS, SU8, glass, etc.), rather than the overall vibration of the channel. On this basis, our simulation can partially solve the difficulty of observing the side of streaming in the channel, and the effect of the upper wall’s position and the microstructure’s machining error angle on the streaming can be investigated, which will provide theoretical guidance for making the side profile as design factors in future research.

Section 2 describes the numerical method in detail, including the model geometry and numerical scheme, governing equations and boundary conditions, and mesh independence test. Section 3 presents the effect of the side profile on streaming including under various boundary layer thickness parameters. The main results are summarized in Section 4.

## 2. Numerical Methods

### 2.1. Model Geometry and Numerical Scheme

**Figure 2:**
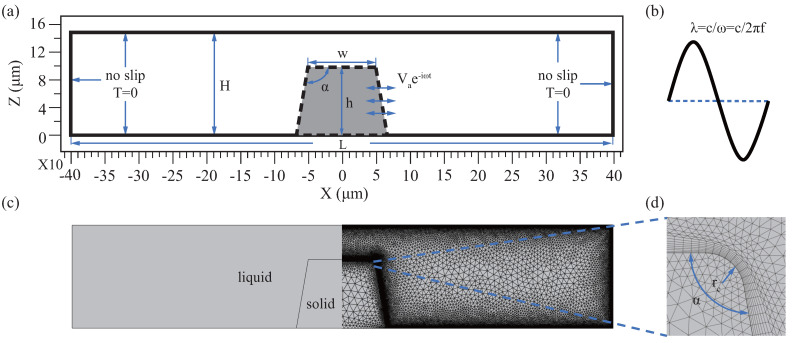
Two−dimensional simulation model is established to analyze the effect of side profile on streaming. (**a**) The model’s geometry and representative parameters as well as the movement and temperature boundary conditions are presented, where the black dotted line is the fluid-solid coupling boundary applied $V_{1}=V_{a}e^{-i\omega t}$. (**b**) The wavelength λ of sound wave propagation in fluid. (**c**) The model is axisymmetric, the left side represents the physical domain including fluids and solids, and the right side represents the mesh situation with (**d**) a magnified view around the tip.

Figure 2a demonstrates the side profile diagram of the microstructure-induced streaming in the channel, which includes the microstructure’s sidewall profile and the channel’s upper wall. Among them, the microstructure sidewall profile includes the profile angle α, width *w*, and height *h*. For the width *w*, the diameter of the micropillars has little effect on the streaming, so we ignore the effect of the *w* parameter and set w≡100μm. For the profile angle α instead of the ideal vertical angle, we select the machining error with ±10${}^{\circ }$, that is, $\alpha \in {\left[80,100\right]}^{\circ }$. For the height *h*, set h={50,75,100,125,150} μm respectively. In additional, we measure the upper wall’s position by introducing parameter *g* (g=H−h) with a range of 5 to 100 μm. Meantime, we set the channel span L=8w to ignore the influence of the channel’s sidewall.

In this study, the numerical model is symmetrical, which includes both fluid and solid domains, as shown in Figure 2c. The fluid domain takes water as the reference medium, and its properties in the reference state ($T_{0}=25$${}^{o}$C, $p_{0}=101$ kPa) are shown in Table 1 [42,43], the solid domain selects SU8. SU8 photosensitive resin, as a conventional microstructure fabrication material, can be used in non-biological and biological species applications [24,44,45]. Of course, the potential toxicity of SU8 is often a worry, but the study has evaluated SU8 biocompatibility in vivo and in vitro. It was found that the polymerized SU8 leaches very little antimony salts, which is lower than the US EPA recommendation (normal physiological conditions), and biocompatibility may be further enhanced by certain surface treatments [46]. The solid domain is introduced to realize fluid-solid boundary coupling, and its material properties do not affect the results. The investigation found that the vibration of the microstructure is caused by the resonance of the excited substrate [30]. Therefore, we assume that the microstructure is rigid, ignoring deformation. The Figure 2c shows the geometry of the numerical model discretization of side profile. To avoid singularities during the calculation, we set the round $r_{c}$ at the apex of the microstructure, as Figure 2d. The numerical model was built with the finite element software COMSOL Multiphysics, and two sets of governing equations were solved based on PT. First, the first-order acoustic field is calculated using the frequency domain thermoviscous acoustics module. Then the second-order streaming field is then calculated by applying the laminar flow module. For specific numerical schemes, refer to Appendix A.1. Table 1 lists the relevant basic and operating parameters.

### 2.2. Governing Equations and Boundary Conditions

Summarizing theoretical research, fluid is governed by three fundamental equations [47,48], including the continuity, the momentum, and the energy equation. Bold and standard font represent vector and scalar, respectively. Ignoring volumetric forces and heat source terms, we simplify the governing equations as follows:

(1) $$\frac{D\rho }{Dt}=-\rho \nabla \cdot V\;,$$

(2) $$\rho \frac{DV}{Dt}=\nabla \cdot P\;,$$

(3) $$\rho \frac{De}{Dt}=\nabla \cdot \left(k_{th}\nabla T\right)+P:E.$$

where *t* is time, ρ is the fluid density, V is the velocity, *e* is the internal energy of the fluid per unit volume, *T* is the temperature, $k_{th}$ is the thermal conductivity, and P and E are, respectively, the stress and strainrate tensors of fluid. The last term on the right-hand side of Equations (3) is the vector simplified expression, representing P:E=∇·(P·V)−V(∇·P).

For Newtonian fluids, P can be expressed by pressure *p* and V, dynamic shear viscosity η, bulk viscosity $\eta_{b}$, and viscosity ratio β ($\beta =\eta_{b}/\eta +1/3$) as follows:

(4) P=−pI+τ,

(5) $$\tau =\eta \left[\nabla V+{\left(\nabla V\right)}^{T}\right]+\left(\beta -1\right)\eta \left(\nabla \cdot V\right)I\;,$$

where I represents the unit tensor, superscript *T* represents the transpose of matrix, and τ is the viscous portion of P.

PT superimposes second-order flow velocities on the first-order acoustic field, where parameters in the fluid can be expressed as the zero (without sound wave), first and second order quantities, marked as subscripts 0, 1 and 2, respectively. For example:

(6) $$\xi =\xi_{0}+\xi_{1}+\xi_{2},\xi_{1}=\varepsilon \tilde{\xi },\xi_{1}=\varepsilon^{2}\tilde{\xi }.$$

where ε is an infinitesimal quantity of the dimensionless, which can be taken as the ratio ζ of the amplitude of the first-order velocity to the velocity of sound [49]. where $\xi_{1}$ can be expressed as ξ1=ReVae−iωt under harmonic vibration.

Without acoustic waves, the parameters of the fluid are considered constant, while micro perturbations occur when the acoustic waves are present. Assuming that the perturbation is linear, all parameters can to be extended to the first order, as $\xi =\xi_{0}+\xi_{1}$. Combining the first law of thermodynamics ($de=Tds+p/\rho^{2}d\rho$, where *s* is the unit mass entropy), and eliminating the zero-order and the first-order higher-order term, the continuity, the momentum, and the energy equation are in the first-order form [50]:

(7) $$\partial_{t}\rho_{1}=-\rho_{0}\nabla \cdot V_{1}\;,$$

(8) $$\rho_{0}\partial_{t}V_{1}=-\nabla p_{1}+\eta \nabla^{2}V_{1}+\beta \eta \nabla \left(\nabla \cdot V_{1}\right)\;,$$

(9) $$\rho_{0}T_{0}\partial_{t}s_{1}=k_{th}\nabla^{2}T_{1}.$$

The zero-order speeds can only have a considerable effect at unreasonably high-speed background flow (up to 1000 mm/s) [51], hence we set $V_{0}=0$. Combined with the thermodynamic state equation ρ=ρ(p,T) and s=s(p,T), whose total differential form is as follows:

(10) $$d\rho ={\left(\frac{\partial \rho }{\partial p}\right)}_{T}dp+{\left(\frac{\partial \rho }{\partial T}\right)}_{p}dT\;,$$

(11) $$ds={\left(\frac{\partial s}{\partial p}\right)}_{T}dp+{\left(\frac{\partial s}{\partial T}\right)}_{p}dT.$$

For the linearization of above equations, the isothermal compression coefficient $k_{t}$, the isobaric thermal expansion coefficient $\alpha_{p}$ and the specific heat capacity $c_{p}$ are introduced, then Equations (10) and (11) can be simplified as:

(12) $$\rho_{1}=\rho_{0}k_{t}\rho_{1}-\rho_{0}\alpha_{p}T_{1}\;,$$

(13) $$s_{1}=\frac{c_{p}}{T_{0}}T_{1}-\frac{\alpha_{p}}{\rho_{0}}p_{1}.$$

where ${\left(\partial \rho /\partial p\right)}_{T}=\rho k_{t},{\left(\partial \rho /\partial T\right)}_{p}=-\rho \alpha_{p}$,${\left(\partial s/\partial p\right)}_{T}=c_{p}/T$,${\left(\partial s/\partial T\right)}_{p}=-\alpha_{p}/\rho$. Substituting Equations (12) and (13) into Equations (7)–(9) and considering the equations to the first-order, Equations (7)–(9) take the form:

(14) $$\partial_{t}\rho_{1}=-\rho_{0}\nabla \cdot V_{1}\;,$$

(15) $$\rho_{0}\partial tV_{1}=-\nabla p_{1}+\eta \nabla^{2}V_{1}+\beta \eta \nabla \left(\nabla \cdot V_{1}\right)\;,$$

(16) $$\rho_{0}c_{p}\partial_{t}T_{1}-\alpha_{p}T_{1}\partial_{t}p_{1}=k_{th}\nabla^{2}T_{1}\;,$$

(17) $$\rho_{1}=\rho_{0}k_{t}\rho_{1}-\rho_{0}\alpha_{p}T_{1}.$$

Zero-order parameters are considered constants and take values in the reference state. Combined with known boundary conditions, such as $p_{1}$ or $V_{1}$, other first-order parameters can be obtained by Equations (14)–(17).

Although the first-order field has been obtained, considering that the NS equation is nonlinear, the parameters need to be extended to the second-order, as $\xi =\xi_{0}+\xi_{1}+\xi_{2}$. For water and most liquids, the thermal effect is small in the first-order field [43]. And the second disturbance part is generally much smaller than the first order, that is, $T_{2}\ll T_{1}$. Therefore, without considering the coupling between $T_{2}$ with $V_{2}$ and $p_{2}$, the energy equation is removed. Extracting the second-order components and ignoring the second-order higher-order terms, Equations (1) and (2) are organized as follows [52]:

(18) $$\partial_{t}\rho_{2}=-\rho_{0}\nabla V_{2}-\nabla \left(\rho_{1}V_{1}\right)\;,$$

(19) $$\rho_{0}\partial_{t}V_{2}=-\nabla p_{2}+\eta \nabla^{2}V_{2}+\beta \eta \nabla \left(\nabla \cdot V_{2}\right)\;-\rho_{1}\partial_{t}V_{1}-\rho_{0}\left(V_{1}\cdot \nabla \right)V_{1}.$$

The second-order velocity is much smaller than the first-order velocity and can usually be ignored. When time-averaged is considered, the first-order velocity is zero, while the second-order is not. When the time-averaged function $\langle A\rangle =1/\tau \int_{0}^{\tau }A\left(t\right)$ is defined, Equations (18) and (19) can be expressed as:

(20) $$\rho_{0}\nabla \cdot \langle V_{2}\rangle =-\nabla \cdot \langle \rho_{1}V_{1}\rangle \;,$$

(21) $$\eta \nabla^{2}\langle V_{2}\rangle +\beta \eta \nabla \left(\nabla \cdot \langle V_{2}\rangle \right)-\nabla p_{2}=\rho_{0}\langle \left(V_{1}\cdot \nabla \right)\nabla V_{1}\rangle +\langle \rho_{1}\partial_{t}V_{1}\rangle .$$

$V_{2}$ is the acoustic streaming velocity, which can be solved by the parameters $V_{1}$ and $\rho_{1}$ obtained in the first-order acoustic field.

The numerical simulation is based on PT, so the boundary condition setting is done in two steps. The initial conditions of the second-order field are achieved by the inheritance of solutions from the first-order field. Our study is based on the assumption that there is no background flow and the first-order acoustic field wall condition is hard wall, refer to Appendix A.2 for details. For the first-order field, we set the “no-slip boundary condition” ($V_{1}=0$) and $T_{1}=0$ respectively, considering the adhesion and no the temperature change on the channel’s walls, as the solid line in Figure 2a. The vibration velocity $V_{1}=V_{a}e^{-i\omega t}$ of all liquid-solid interfaces is satisfied, including the top of the profile, where the velocity amplitude $V_{a}=2\pi fd_{0}$, as the dotted line in Figure 2a. The vibration direction is parallel to the x-axis. To simplify, we choose the linear vibration mode parallel to the x-axis, which can be achieved by excitation at a specific frequency [32] or by placing the chip on a piezoelectric actuator [53]. For second-order fields, the temperature remains constant, so the temperature condition can be ignored, refer to Appendix B.1. Set only velocity conditions for all fluid boundaries, no slip conditions ($V_{2}=0$).

### 2.3. Mesh Independence Test

Mesh independence test is required in numerical discreteness to obtain the optimal mesh. To reduce computation, fluid domain mesh is divided by region, including high gradient regions near the wall and bulk domain region. For high gradient regions, We set multiple the boundary layer mesh and measure the maximum element size length $d_{mesh,db}$ by boundary layer thickness δ. For bulk domain region, the propagation of acoustic waves in the fluid causes the medium’s density change, whose periodic length is defined as the wavelength λ, as shown in Figure 2b. So the bulk domain region maximum element size $d_{mesh,dk}$ by λ. To get the optimal mesh of high gradient regions and bulk domain regions, we set up seven sets of meshes, see the Appendix A.3. The solid domain mesh uses the physics-controlled mesh.

**Figure 3:**
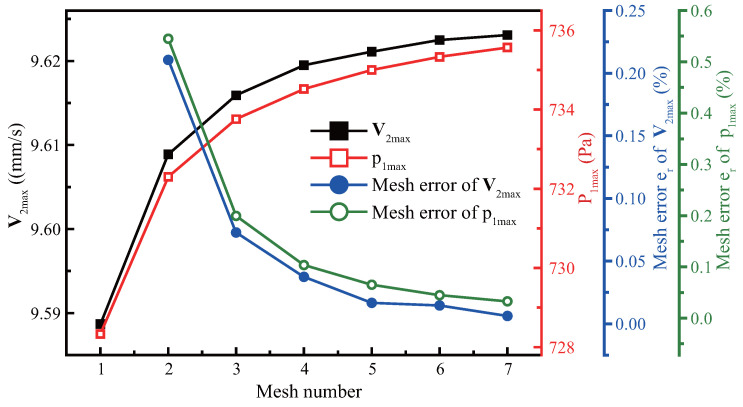
Mesh independence test.The first-order field $p_{1max}$ (red line) and the second-order field $V_{2max}$ (black line) change with the mesh densification. The blue and green line represent the mesh error of $V_{2max}$ and $p_{1max}$ as the mesh size decreases respectively.

The evaluation of the mesh independent test was performed at the basic boundary layer thickness (when f=10kHz, η=0.89mPa·s). For the first-order and second-order fields, we choose the maximum first-order pressure $p_{1max}$ and the maximum second-order velocity $V_{2max}$ to evaluate the grid convergence. We set the relative mesh convergence error $e_{r}$ using Equations (22) as follows:

(22) $$e_{r}=\frac{\left|R_{,cur}-R_{,pre}\right|}{R_{,pre}}\times 100\%.$$

where $R_{,cur}$ represents the result calculated under the current mesh and $R_{,pre}$ is the result of the previous coarse mesh.We use $V_{2max}$ and $p_{1max}$ instead of *R* to calculate the relative error respectively. Figure 3 demonstrates that with the continuous refinement of the mesh, $p_{1max}$ and $V_{2max}$ tend to constants, and the relative error $e_{r}$ parameters of $V_{2max}$ and $p_{1max}$ are less than 0.05% at the sixth mesh. To ensure accuracy we chose the sixth mesh ($d_{mesh,db}=\delta /6$, $d_{mesh,dk}=\lambda /12$), which is selected for all subsequent research cases.

## 3. Results and Discussion

In this study, the effect of side profile on acoustic streaming is analyzed by numerical simulation. Compared with theoretical calculation, it is not limited by geometric shape. Currently, theoretical calculations of two-dimensional models are mostly circular, but its isotropic can be simplified by polar coordinates. Applying above model to circular, the maximum streaming velocity is slightly higher than the theoretical calculation results [53]. This is because our model considers the compressibility and thermal viscosity, increasing the nonlinearity of the acoustic disturbance fluid. The details are referred to Appendix A.4. The article introduce three parameters including $V_{2max}$, $S_{A}$, and model to describe the effect on streaming characteristics.The model is defined as the shape, number, and location of the vortices. The $S_{A}$ is defined as the area where the streaming velocity is not less than A $\times V_{2max}$, for example $S_{0.9}=\int \left(V_{2}\geq 0.9\times V_{2\max}\right)ds$. Our research focuses on the streaming which is built on the basis of the first-order field refering to the Appendix B.1.

### 3.1. The Effect of Side Profile Parameters

In this section, the effect of the sidewall angle α and the gap *g* on the streaming is mainly investigated. In addition, the microstructure’s sidewall profile also contains the width *w* and heigh *h* which is worth considering for boundary streaming. Under the perturbation theory, the previous research has shown that the diameter of the micropillars has little effect on the induced streaming [39]. Therefore, we ignore the effect of weight *w* and set *w* ≡ 100 μm. Subsequently, this article only focuses on *h*, to investigate the effects of α and *g* on the streaming under different *h*. For this section, we set basic parameters as: f=10 kHz, η=0.89 mPa·s (δ=5.3 μm).

#### 3.1.1. The Effect of Gap

**Figure 4:**
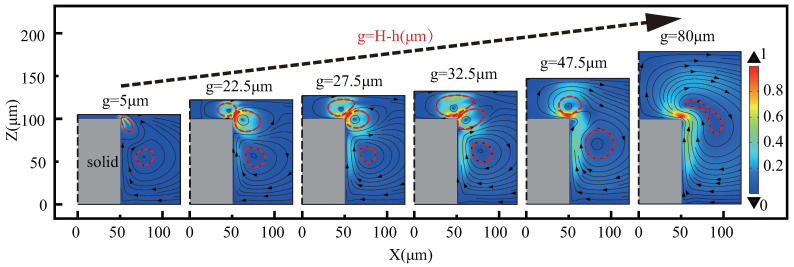
Changes in the acoustic streaming patterns with the gap. The vortexes are all reverse−symmetrical, such as red dotted lines, and show only half, with black dotted lines being the axis of the symmetry. Among them, the high-speed vortex is a vortex with a velocity greater than 0.5, otherwise it is a low-speed vortex. The legend of each cloud atlas divided by the respective maximum streaming speed.

**Figure 5:**
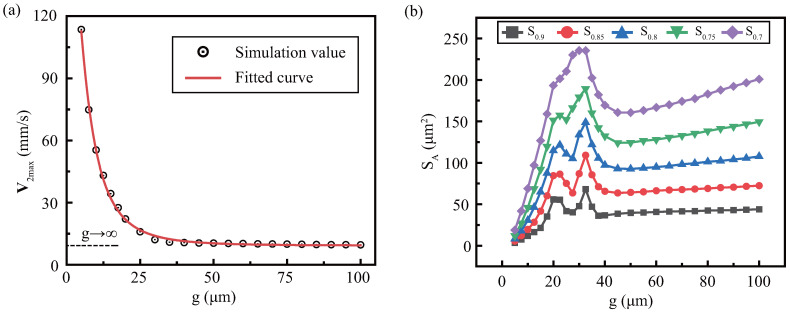
The gap effect on streaming is revealed by quantitative parameters: (**a**) $V_{2max}$ changes obviously, where the black discrete point is the numerical value, and red is the fitting curve when it tends to infinity, which is approximately equal to $V_{2max}^{\infty }$, such as the black dotted line. (**b**) Effective areas $S_{A}$ changes are complex, including $S_{0.9}$, $S_{0.85}$, $S_{0.8}$, $S_{0.75}$ and $S_{0.7}$. Constant parameter: h=100 μm, $\alpha =90^{\circ }$, f=10 kHz, η=0.89 mPa·s.

The streaming pattern consists mainly of reverse symmetrical vortexes, with the right-half shown in Figure 4. Employing local enlargement maps near microstructures, we discovers that different positions (e.g., different *g*) of the upper wall differ in number, position, and intensity of vortexes. Classified by the number and shape of the vortexes, the mode can be divided into five phases as the gap increases: space limitation, high-speed vortexes development, maximum action area development, high-speed vortexes fusion, and all vortexes fusion. Due to the space constraints of g=5∼22.5 μm, the range of vortex close to the upper wall surface is small, continuously expands, and another high-speed vortex also slowly expands. When g=22.5∼27.5 μm, the two high-speed vortexes all grow at this phase, but grow faster near the upper wall, and eventually reach equality. When g=27.5∼32.5 μm, the high-speed vortex near the upper wall expands, resulting in a maximum action area, refer to Figure 5b. When g=32.5∼47.5 μm, the high-speed vortex near the upper wall expands and another high-speed vortex shrinks, and finally forms a high-speed vortex. When g=47.5∼80 μm, all vortexes merge into one pair with an increase in gap, similar to top-view mode.

Figure 5a shows that $V_{2max}$ generally decreases with *g* increases, showing a tendency to initially decrease rapidly and stabilize slowly. Furthermore, using the curve fitting, we found that the $V_{2max}$ with increasing gap tends to be constant, as the red solid line. Subsequently, $V_{2max}$ by solving the curve limitation is about 9.3 mm/s when g=∞, which is denoted as $V_{2max}^{\infty }$ as the black dotted line.

When considering the upper wall, streaming enhances especially at small gap, and the effective areas also change significantly, see Figure 5b. To normalize the streaming range, effective regions $S_{A}$ are used, which also applies to future studies. The figure lists the different effective areas $S_{0.9}$, $S_{0.85}$, $S_{0.8}$, $S_{0.75}$, and $S_{0.7}$, and found the rules to be similar. They are content with a rapid rise first, then a peak volatility, and then a slowdown after a certain decline. In particular, peak fluctuations in different $S_{A}$ have a maximum extreme point with about the same *g*. The *g* can achieve the maximum effective area of streaming, called optimal gap. When comparing different $S_{A}$, the areas in small gap are small and approximately the same, without obvious delamination, but the delamination becomes obvious as gaps increase.

The reduction in $V_{2max}$ indicates that the presence of upper wall increases streaming intensity. However, as the gap increases, the dissipation viscosity near the upper wall decreases and the increase effect weakens. Note that this decline is not merely linear, which may be influenced by spatial effects. When the gap is small, the main viscosity dissipation energy is trapped in a narrow space. However, as the gap increases, the dispersion area increases rapidly, leading to energy dispersion (as Figure 4). At present, the gap in the study of vibration-induced streaming is limited to a specific range. However, the increase of flow velocity caused by the decrease of clearance is obvious, which helps to deduce the subsequent acoustic flow research under small gap. Furthermore, under small gaps, effective areas are not clearly delaminated due to limited space. For optimal gaps, the gap can be considered to provide sufficient space, while maintaining sufficient viscous dissipation near upper wall. If the gap is extended further to g=100μm, the effective area and streaming mode are usually stable, and the relative maximum speed difference is $\varepsilon =V_{2\max}/V_{2\max}^{\infty }=3.35\%$. In this case, the effect of the upper walls is small, and it can be assumed that there is no upper wall.

#### 3.1.2. The Effect of Sidewall Angle

As the Section 1, the manufacturing process of lithographic-related microstructures produces sidewall profile angles α which are affected by the light source, exposure method, and type of photoresist. In this article, the sidewall profile angle is set to $90\pm 10^{\circ }$. Through the study in the Section 3.1.1, we set g≡100 μm to ignore the upper wall and only study the sidewall angle.

**Figure 6:**
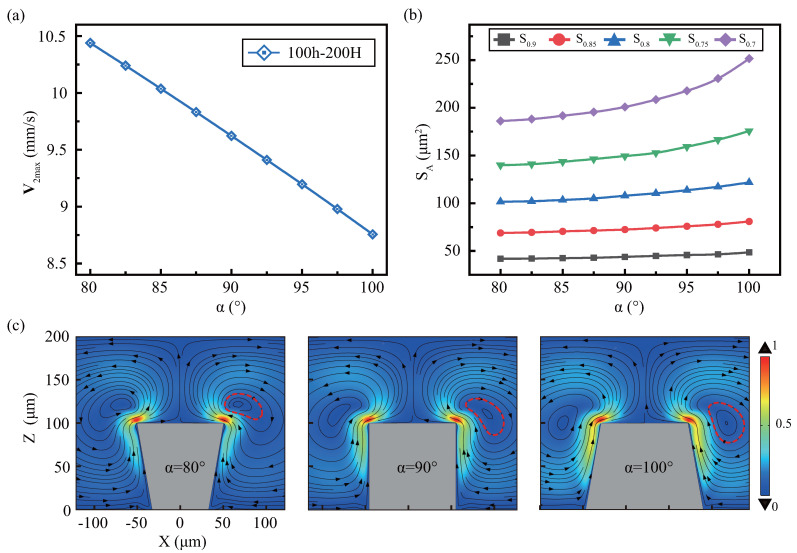
The effect of the microstructure sidewall profile’s angle α on the streaming. Under fixed microstructure conditions of h=100 μm and H=200 μm, the angle α effect on (**a**) maximum speed, (**b**) effective areas, and (**c**) mode where the red dotted line represents the streaming was studied. The legend of each cloud atlas divided by the respective maximum streaming speed.

Figure 6a demonstrates that the $V_{2max}$ decreases approximate linearly with α increase and the effective areas are reversed. As the angle increases, different effective areas expand further, and each effective area’s stratification has less influence by the angle, show in Figure 6b. As show in Figure 6c, the sidewall angle has little impact on streaming mode. The overall performance is a pair of anti-vortex, the angle increases, the vortex intensity decreases, the area increases, and the vortex position changes slightly.

According to previous analyses, angle reduction increases $V_{2max}$ but reduces the effective areas. This indicates that angle sharpness increases streaming, but the effective area is limited. It is because structural sharpening concentrates energy distribution regions at the tip, while structure passivation expands the distribution regions and disperses energy.

#### 3.1.3. The Effect of Height

**Figure 7:**
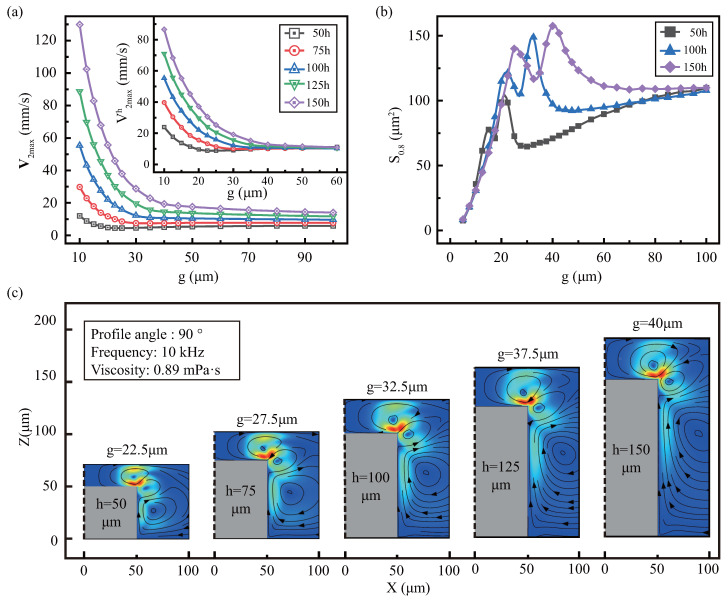
The effect of the gap under various *h* on the streaming: (**a**) the maximum streaming velocity, where the altitude equivalent velocity is displayed in the inset. (**b**) effective area $S_{0.8}$. (**c**) streaming patterns under optimal gap, in which the black dotted line is the symmetry axis, and the gray is the microstructure.

As an important factor in boundary streaming, height is worthy of attention. In this section, we investigate the effects of sidewall profile angle α and upper wall gap *g* on streaming at various heights. We set h={50,75,100,125,150} μm is added, and its aspect ratio is 0.5, 0.75, 1, 1.25, 1.5, respectively. Figure 7 shows the effect of *g* on the streaming under various heights. As show in Figure 7a, $V_{2max}$ is positively associated with *h* under the same *g*. In addition, we set height-normalized equivalent velocity $V_{2\max}^{h}=100\times V_{2\max}/h$, when the gap (≥50 μm) is large, the $V_{2\max}^{h}$ under various *h* is approximately equal. But when the gap is small, the $V_{2\max}^{h}$ generated by the higher height is also larger. This indicates that if the gap is large, $V_{2\max}^{h}\propto h$ and small, $V_{2\max}^{h}\propto h^{2}$.

Figure 7b describes the effect of the gap under various *h* on the effective area. We combine the respective effective area $S_{0.8}$ together, selecting various heights at intervals to get a clear expression, and the complete results can be found in Appendix B.2. The influence of *h* on $S_{0.8}$ is small whether the gap is large or small, but if g=15∼45 μm, *h* has a large influence, causing a peak fluctuation in the effective area. Figure 7c shows the optimal gap mode diagram under different heights, with half of the symmetrical diagram selected. It was found that higher *h* not only generates larger effective areas, but also requires larger optimal clearance. Furthermore, with the change of the gap under various *h*, the model all have five stages, as in Section 3.1.1, only the range of the stages is changed.

The higher *h* has more viscous dissipation area, which will improve the strength and effective area of streaming. When the gap is small, $V_{2max}$ is not only affected by the height’s positive correlation, but also by the spatial limitation of the upper wall, which amplifies the positive correlation. However, the space limitation hinders the expansion of the effective area, making $S_{0.8}$ is roughly the same for different *h*. When g=15∼45 μm, the higher the height, the larger the effective area, due to the more the viscous dissipation area on the boundary layer. It is worth noting that the optimal gap is larger for higher *h*, because more viscous dissipation requires more space to release. Thus, the effective area is affected by gap and height.

**Figure 8:**
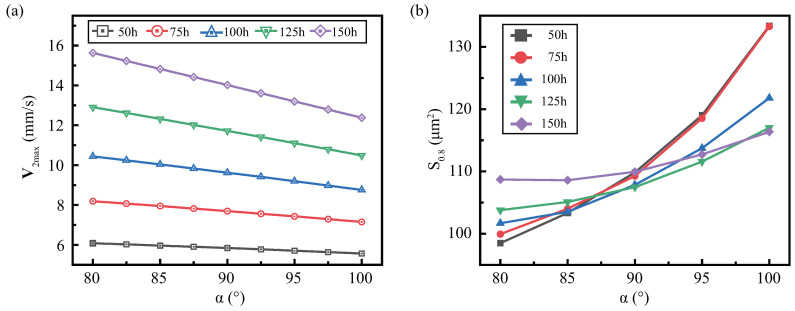
The effect of the sidewall angle under various *h* on the streaming: (**a**) maximum streaming velocity (**b**) effective area $S_{0.8}$. Constant parameters: f=10 kHz, η=0.89 mPa·s.

When discussing the effect of α on the streaming under various h, we fixed the gap g=100μm (H−h=100 μm). Since the mode change was small, it is not discussed in this article. And the general rules of $V_{2max}$ and $S_{A}$ are similar for various height, but have small differences, as shown in Figure 8. Figure 8a shows that $V_{2max}$ decreases more steeply with decreasing α at higher *h*, which is related to the viscous boundary layer. Figure 8b shows the impact of sidewall angles for various heights on the effective area and finds that the slope of $S_{0.8}$ with α is inversely proportional to height. This is since when α<90${}^{o}$, the higher height collects more dissipated energy at the tip, and its effective range is larger. When α>90${}^{o}$, the larger height, the more the viscous dissipation in the sidewall of microstructure, but the more dispersed the viscous dissipation region, resulting in a slower increase.

### 3.2. The Effect of Boundary Layer Thickness Parameters

The streaming is controlled not only by side profile (geometry) but also by frequency and viscosity related to δ. To broaden the applicability of study, we select various boundary layer thickness parameters to investigate the effects of *g* and α. We set the parameters $f=\left\{f_{0.5},f_{0.75},f_{1},f_{1.25},f_{1.5}\right\}$, $\eta =\left\{\eta_{0.5},\eta_{0.75},\eta_{1},\eta_{1.25},\eta_{1.5}\right\}$, where the subscripts represent the multiplication factor of the basic parameters. In this paper, the boundary layer thickness parameters have little effect on mode, so the effects on mode are not considered.

#### 3.2.1. The Effect of Frequency

**Figure 9:**
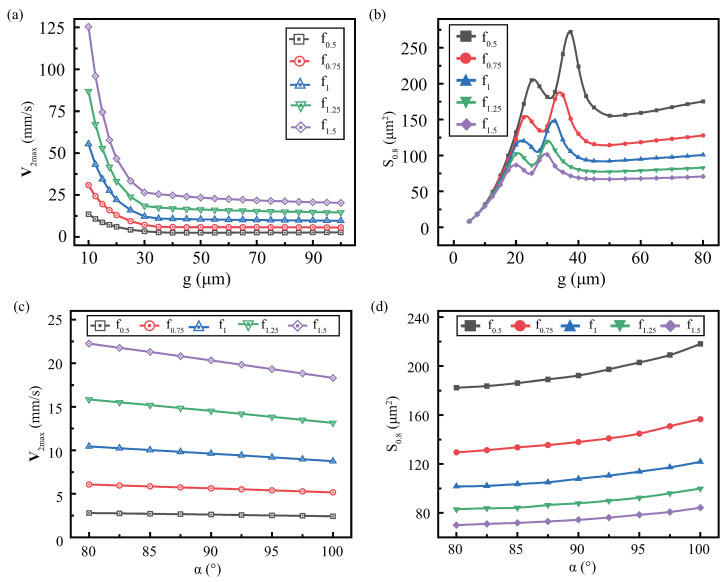
Effect of side profile parameters on streaming under viscous frequency. There are the effect of the gap on the maximum streaming velocity (**a**) and effective area (**b**), as well as the effect of the sidewall angle on the maximum streaming velocity (**c**) and effective area (**d**).

In this section we set η≡0.89 mPa·s and find that the effects of *g* and α on streaming under the various frequency, similar to basic frequency (in Section 3.1.1 and Section 3.1.2). But the differences under various frequencies are obvious, details as follow:

Figure 9a shows the effect of *g* on $V_{2max}$ with various *f*, where h=100 μm, α=90${}^{o}$. When *g* is the equal, $V_{2max}$ increased with increasing frequency. The equivalent velocity of frequency $V_{2\max}^{f}=V_{2\max}/{\left(f/f_{1}\right)}^{2}$ is introduced. It is found that the equivalent velocity from various frequencies under all gaps is approximately equal, as Appendix B.2. This indicates that $V_{2max}$ is proportional to the square of the frequency, not affected by the gap. In other words, the small gap does not amplify the enhancement effect of high frequencies, which is different from height. This article adopts a fixed vibration displacement amplitude $d_{0}$, so the velocity amplitude $V_{a}$ is proportional to the frequency *f*, $V_{a}=2\pi d_{0}f$. Thus, $V_{2max}$ is also proportional to the square of $V_{a}$ [54], $V_{2\max}=\theta V_{a}^{2}=\theta {\left(2\pi d_{0}f\right)}^{2}$. Substituting the above equation into the equivalent velocity of frequency, get:

(23) $$V_{2\max}^{f}=\theta \times {\left(2\pi d_{0}f_{1}\right)}^{2}=\theta \times O.$$

where *O* is the operational coefficient. For the fixed operational parameters, *O* is a constant. $V_{2\max}^{f}$ declined rapidly first and then flattened, which indicates that θ is affected by the gap.

Figure 9b shows the effect of gap on the effective area at various frequencies, where the effective area $S_{0.8}$ at each frequency is collected and combined. If the gap is large (the upper wall can be ignored), the effective area is approximately inversely proportional to the frequency. This can be attributed to the sound wave propagating in the fluid, whose wavelength is $\lambda =c_{0}/\omega$. The lower the frequency, the larger the wavelength, the larger the disturbance area and the larger the effective area. As the gap decreases, the $S_{0.8}$ of each frequency appears peak fluctuations, and the ordinate of the peak is inversely proportional to the frequency. Moreover, due to the combined effect of wavelength and spacing, the abscissa (optimal gap) of the peak also increases in a small range with the frequency decreases.

As shown in Figure 9c, $V_{2max}$ satisfies the decrease as α increases, and $V_{2max}$ is proportional to frequency under the equal α. The $V_{2\max}^{f}$ at each frequency is approximately equal and decreases with the increase of angle, which indicates that θ and the sidewall angle α are negatively correlated, as Appendix B.2. For $\alpha \in {\left[80,100\right]}^{\circ }$, $S_{0.8}$ is approximately inversely proportional to the frequency, as Figure 9d. This also contributes to wavelength, similar to large gaps without spatial limitation.

#### 3.2.2. Influence of Viscosity

**Figure 10:**
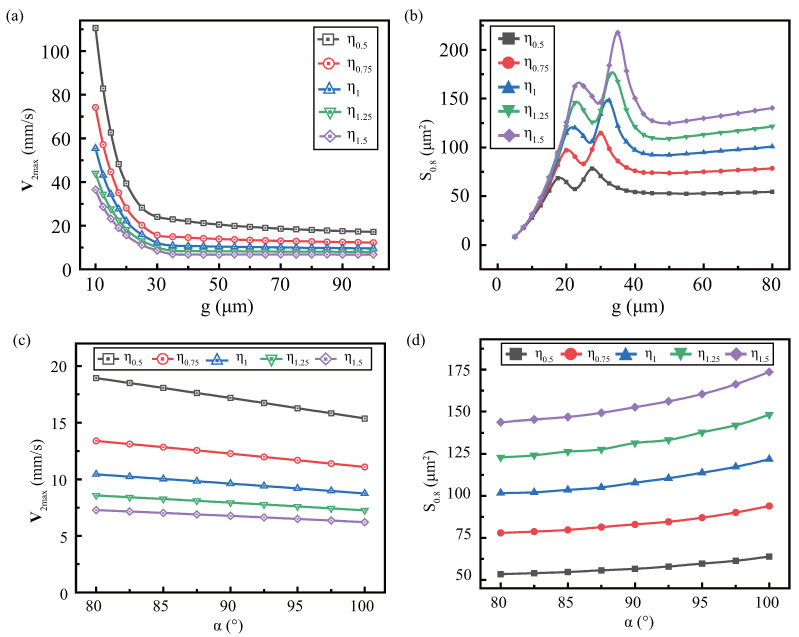
Effect of side profile parameters on streaming under viscous viscosity. There are the effect of the gap on the maximum streaming velocity (**a**) and effective area (**b**), as well as the effect of the sidewall angle on the maximum streaming velocity (**c**) and effective area (**d**).

This section will consider another boundary layer thickness factor, viscosity η. we set f≡10 kHz and find that the effects of *g* and α on streaming under the various viscosity, similar to basic viscosity. But the differences under various viscosities are obvious, details as follow:

From Figure 10a, when *g* increases, $V_{2max}$ decreases rapidly and then tends to stabilize slowly, which is the same as constant viscosity. But the maximum velocity at the equal gap is inversely proportional to viscosity. Figure 10b shows the effect of the effective area $S_{0.8}$ with the gap at different viscosities. When *g* is small, the $S_{0.8}$ is almost equal at different viscosities, which is attributed to the limited space that restricts the development of flow. For large *g*, $S_{0.8}$ is proportional to η, because the effect of the upper wall weakens, and the viscosity dominates. Moreover, $S_{0.8}$ still has a peak fluctuation at medium gap. the ordinate and abscissa of peak are proportional to the viscosity, which is just opposite to the frequency. It is worth noting that the small difference in the abscissa of peak is the combined effect of viscosity and gap.

Figure 10c shows the effect of α on sound flow at different viscosity. $V_{2max}$ generally decreases with the increase of α, and $V_{2max}$ is inversely proportional to η when α is equal. Figure 10d is a combination of $S_{0.8}$ extracted from each viscosity. it is found that $S_{0.8}$ is proportional to the viscosity at the same α. This indicates that the higher viscosity, the lower the intensity of disturbance, and the greater the range of disturbances. Therefore, when the viscosity increases, $V_{2max}$ decreases, and the effective area increases.

## 4. Conclusions

Based on perturbation theory, we develop a 2D simulation model to analyze the effects of side profile and boundary layer thickness on acoustic streaming. Using the coupling boundary of fluid-soild, we define three parameters to characterize the streaming, including the mode, maximum streaming velocity, and effective area. Review and summarize the main conclusions as follow:

For the side profile parameters, our model predicts that the presence of the upper wall makes the streaming more intense and the pattern more complex. $V_{2max}$ increases compared to ignoring the upper wall, the extent of the increase depends on the viscous dissipation and space near the upper wall. Consequently, the streaming velocity in the small gap is stronger. At the same time, the existence of the upper wall enriched the types of patterns, which can be divided into 5 phases according to number and shape of the vortexes. For another sidewall profile parameter, the sidewall angle α also has a obviously impact except the pattern. The sharper the structure yields a larger $V_{2max}$, while the effective area is smaller. Subsequently, we investigate the effect of side profile parameters on streaming under various heights. When the gap is large, $V_{2max}$ is mainly controlled by height and is proportional to the height approximation. This rule also holds true when investigating the effect of α on streaming. But, at small gap, $V_{2max}$ is controlled by both the space and height. The effects of gap and angle on the effective area are similar at various heights, but the higher *h*, the greater the optimal gap is required.

For the boundary layer parameters, the effect on the mode is small, but the effect on the quantitative parameters is obvious. The effect of viscosity and frequency on the streaming is opposite. The higher the viscosity the lower the perturbation intensity of the streaming, but the wider the perturbation region, which is the opposite of the frequency. $V_{2max}$ is inversely proportional to the viscosity when the gap is large and when the α is studied. But proportional to the square of the frequency, $V_{2\max}=\theta V_{a}^{2}=\theta {\left(2\pi d_{0}f\right)}^{2}$, where θ may be related to the side profile. When the gap is small, $V_{2max}$ and effective area are greatly affected by space. Furthermore, the optimal gap of $S_{0.8}$ yields small deviations due to the difference of frequency and viscosity, and the streaming with lower frequency and higher viscosity requires more space to develop.

In conclusion, this study focuses on the effect of side profile on streaming. We use no-slip boundary condition and sidewall angle to introduce upper wall and machining error respectively, which will be closer to reality. Moreover, it also provides a theoretical basis for designing the upper wall’s position to adjust the streaming. Subsequently, this study explored the effect of side profile under various boundary layer thicknesses to expand the adaptable range. Our findings may optimize various applications of microstructure-induced streaming, such as particle manipulation, cell rotation, and micromixing. Sharper sidewall profile angles result in greater streaming velocity at the tip, promising fast target manipulation. Compared with no upper wall, the mode of the streaming is more complicated existing multiple pairs of vortices under a certain gap, which will improve the micro-mixing efficiency.

## Figures and Tables

**Table 1:** Constitutive and operational parameters.

Parameter	Value	Units
Density, $\varrho_{0}$	997	kg/m^3^
Speed of sound, $c_{0}$	1496.73	m/s
Dynamic shear viscosity, η	0.89	mPas
Bulk viscosity, $\eta_{b}$	2.47	mPas
Thermal conductivity, $k_{th}$	0.6075	W/m·K
Specific heat capacity, $c_{p}$	4181.5	J/kg·K
Thermal expansion coefficient, $\alpha_{p}$	2.57 × 10^−4^	1/K
Compressibility coefficient, $k_{t}$	448	T/Pa
Gap, *g*	5∼100	μm
Height of the channel, *h*	50/75/100/125/150	μm
Span of the channel, *L*	800	μm
Height of the microstructure, *H*	H=h+g	μm
Span of the microstructure, *w*	100	μm
Profile angle of microstructure, α	80∼100	°
Round radius of apex, $r_{c}$	0.05	μm
Forcing frequency, *f*	5∼15	kHz
Displacement amplitude, $d_{0}$	1	μm

**Figure A1:**
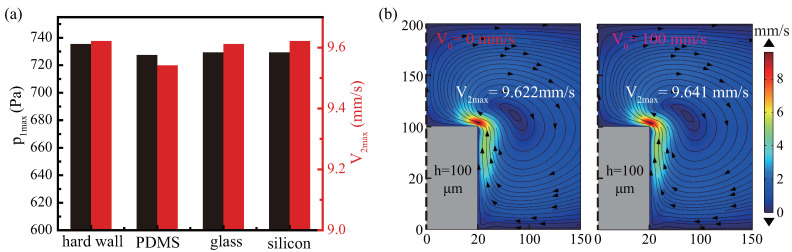
Simplification of Boundary Conditions. (**a**) Comparing the effects of first-order acoustic field wall conditions including hard and impedance wall on $p_{1max}$ and $V_{2max}$. (**b**) Comparing the effects of different background flow velocities (0 and 100 mm/s) on streaming. Where h=100 μm, H=200 μm, f=10 kHz, η=0.89 mPa.

**Figure A2:**
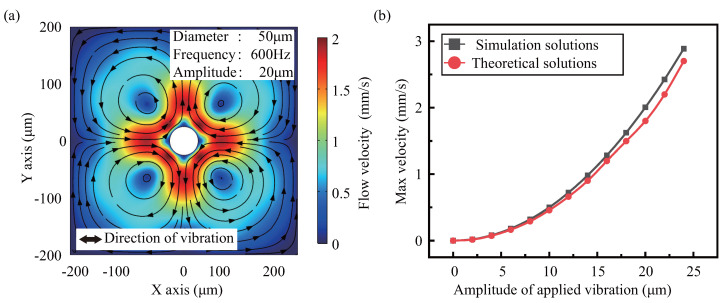
Numerical simulation VS Theoretical calculation. (**a**) Two–dimensional distribution of the flow velocity obtained by simulation. (**b**) Maximum velocity of simulation and theoretical solutions under different vibration amplitudes. The red line and black line represent theoretical solutions and simulation solutions respectively.

**Figure A3:**
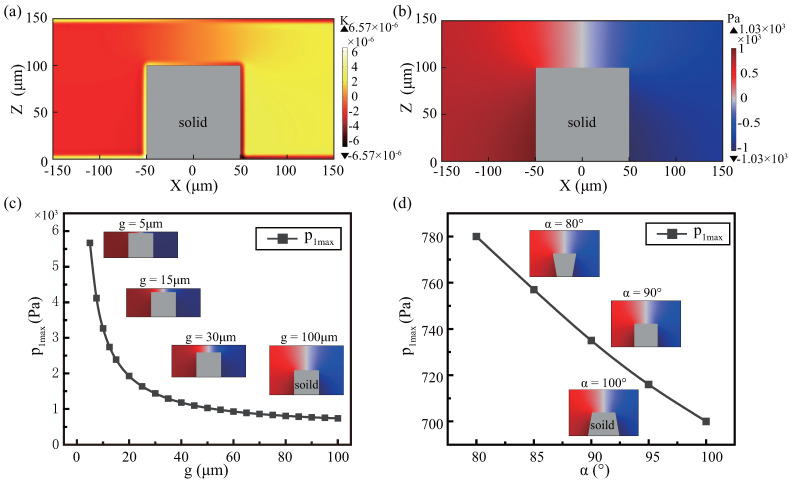
First-order acoustic field simulation.Microstructural vibrations can cause changes in the temperature (**a**) and sound pressure (**b**) of the fluid in the channel, where the gap is fixed at 50 μm. In addition, the side profile can change the size and distribution of the sound pressure, including the gap (**c**) and the side angle (**d**), where the inset shows the sound pressure distribution.Where f=10 kHz, η=0.89 mPa.

**Figure A4:**
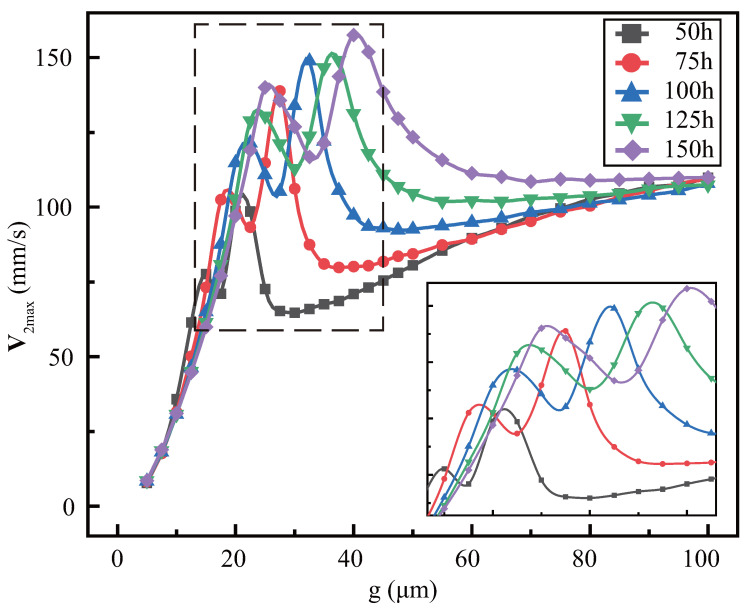
The effect of gap on maximum streaming velocity under five types of heights. Local amplification in the dotted line frame, see inset.

**Figure A5:**
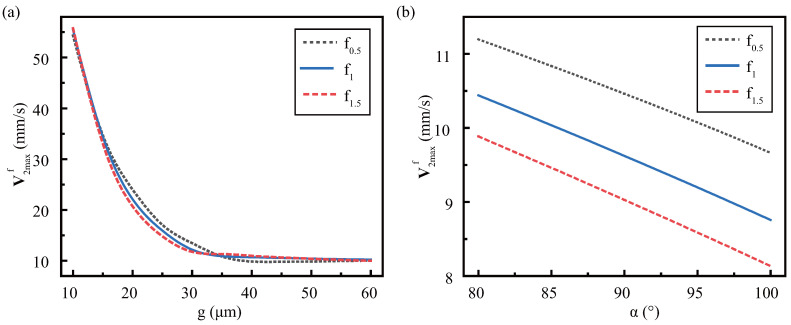
The effect on frequency equivalent velocity by side profile parameters: (**a**) gap *g* and (**b**) sidewall angle α.

## Data Availability

The data that support the findings of this study are available within the article.

## Appendix A. Supplementary of Simulation

### Appendix A.1. Numerical Scheme

Based on PT, we use the finite element software COMSOL to solve step by step, including the first-order acoustic field and the second-order streaming field.

The initial state selects the liquid’s reference state. The first-order acoustic field is calculated using the “Thermoviscous Acoustics, Frequency Domain” module. If A satisfies the harmonic, the partial derivative in the time domain is equal to multiplying iωA in the frequency domain, then Equations (14)–(17) can be converted into:

(A1) $$i\omega \rho_{1}=-\rho_{0}\nabla \cdot V_{1},$$

(A2) $$i\omega \rho_{0}V_{1}=-\nabla p_{1}+\eta \nabla^{2}V_{1}+\beta \eta \nabla \left(\nabla \cdot V_{1}\right),$$

(A3) $$i\omega \rho_{0}c_{p}T_{1}-i\omega \alpha_{p}T_{1}=k_{th}\nabla^{2}T_{1},$$

(A4) $$\rho_{1}=\rho_{0}k_{t}\rho_{1}-\rho_{0}\alpha_{p}T_{1}.$$

where the angular frequency ω=2πf. To obtain the time average in second-order streaming field, it is calculated by the “Laminar Flow, Steady State” module,

(A5) $$\rho \left(V_{2}\cdot \nabla \right)V_{2}=\nabla \cdot \left[-p_{2}I+K\right]+F,$$

(A6) $$K=\eta \left[\nabla V_{2}+{\left(\nabla V_{2}\right)}^{T}\right],$$

(A7) $$\rho \nabla \cdot V_{2}=0,$$

(A8) $$F_{2}=\langle \rho_{1}\frac{\partial V}{\partial t}\rangle +\rho_{0}\langle \langle V\cdot \nabla \rangle V\rangle .$$

where $F_{2}$ is the time-averaged “body force” over a period, and K represents the viscous shear stress matrix associated with streaming [37]. Meanwhile, weak contribution terms are added in the second-order field to enhance computational stability [43].

### Appendix A.2. Simplification of Boundary Conditions

The wall boundary condition of the first-order sound field is the hard wall. The reflection boundary conditions for the propagation of sound waves between different materials depend on the difference in acoustic impedance. Common materials for manufacturing microchannels are PDMS, glass, and silicon whose acoustic impedances are 0.99, 17, and 20 MRayls, respectively. The sound in the flow field mainly comes from two parts, one is generated by the piezoelectric transducer and passed into the fluid through the air and the channel successively, and the other is generated by the vibration microstructure as the sound source. In the former method, the propagation of sound from the air into the channel will be mostly blocked, because the acoustic impedance of the air is much lower than the channel material, so the channel walls can be assumed to be hard walls. In the latter method, the sound generated by the vibration of the microstructure may escape through the fluid to the channel wall. In the latter method, the sound generated by the vibration of the microstructure may escape through the fluid to the channel wall. We set up a variety of impedance walls where the bottom maintains glass which is usually used as the substrate compared with the hard wall, and discover the sound pressure and streaming velocity decreased slightly, as shown in the Figure A1a. It shows that the sound wave generated is almost completely reflected on the channel wall, so we assume that the first-order sound field boundary is a hard wall.

Usually, the sample supply on the microfluidic chip is precisely controlled using micro syringe pumps. The background flow velocity of the microchannel in the chip is generally lower than 100 mm/s, so we set the background flow $V_{0}=100$ mm/s. Compared with no background flow, the background flow in the microfluidic chip flow rate range has less effect on the streaming, including mode and velocity, as Figure A1b. So, we ignore the background flow to simplify the model.

### Appendix A.3. Mesh Size in Fluid Domain

Mesh size is an important factor to ensure the accuracy of COMSOL finite element analysis. The larger the mesh size, the greater the discrete error. With the decrease in size, the calculation accuracy is ensured, but the demand for computing resources increases. Therefore, it is necessary to find a suitable size. To reduce computation, we divide meshes by region. The boundary layer mesh with the maximum size $d_{mesh,db}$ is set in the fluid region near the boundary layer. For the other fluid domain, the acoustic wave propagates at wavelength λ with a small velocity gradient, so we set the maximum size $d_{mesh,dk}$. We set up seven different meshes to explore the optimal size. Table A1 lists the details of the meshes whose size decreases in order.

**Table A1:** Mesh size in fluid domain.

Type	Bulk Mesh $d_{\mathrm{mesh},\mathrm{dk}}$	Boundary Mesh $d_{\mathrm{mesh},\mathrm{db}}$
1	λ/2	δ/1
2	λ/4	δ/2
3	λ/6	δ/3
4	λ/8	δ/4
5	λ/10	δ/5
6	λ/12	δ/6
7	λ/14	δ/7

### Appendix A.4. Numerical Simulation vs. Theoretical Calculation

Based on the incompressible fluid with constant viscosity, the theoretical calculation obtains the flow field by solving the stream function. The stream function ψ can be expressed as:

(A9) $$\nabla^{4}\psi -\frac{1}{\eta }\frac{\partial }{\partial t}\psi =\frac{1}{\eta }V\cdot \nabla \left(\nabla^{2}\psi \right)$$

Limited by the difficulty of solving, the theoretical method can only solve the flow field around the simple shape. Takeshi et al [53] assumed a two-dimensional system in a cylindrical coordinate system to solve the vibration-induced flow velocity around a single micropillar. The steady-state term of the stream function $\psi_{st}^{\left(1\right)}$ can be written as follows:

(A10) $$\begin{array}{ccc} \psi_{st}^{1}\left(r\right)= & & \pm \left[r^{4}\left(\frac{1}{48}\int_{a}^{r}\frac{1}{x}\rho \left(x\right)dx+c_{1}\right)+r^{2}\left(-\frac{1}{16}\int_{a}^{r}x\rho \left(x\right)dx+c_{2}\right)\right]\\ & & +\left(\frac{1}{16}\int_{a}^{r}x^{3}\rho \left(x\right)dx+c_{3}\right)+\frac{1}{r^{2}}\left(-\frac{1}{48}\int_{a}^{r}x^{5}\rho \left(x\right)dx+c_{4}\right), \end{array}$$

(A11) $$c_{1}=-\frac{1}{48}\int_{a}^{\infty }\frac{1}{x}\rho \left(x\right)dx,$$

(A12) $$c_{2}=\frac{1}{16}\int_{a}^{\infty }x\rho \left(x\right)dx,$$

(A13) $$c_{3}=\frac{a^{4}}{16}\int_{a}^{\infty }\frac{1}{x}\rho \left(x\right)dx-\frac{a^{2}}{8}\int_{a}^{\infty }x\rho \left(x\right)dx,$$

(A14) $$c_{4}=-\frac{a^{6}}{24}\int_{a}^{\infty }\frac{1}{x}\rho \left(x\right)dx+\frac{a^{4}}{16}\int_{a}^{\infty }x\rho \left(x\right)dx.$$

where *r* and θ are the radius and angle in polar coordinates, and *a* is the diameter of the micropillar. The flow velocity V can be expressed as:

(A15) $$V_{r}=-\frac{1}{r}\frac{\partial \psi_{st}^{\left(1\right)}}{\partial \theta },\;V_{\theta }=\frac{\partial \psi_{st}^{\left(1\right)}}{\partial r}$$

When the diameter of the 50 μm cylinder applies 20 μm amplitude at 600 Hz, the velocity field in the fluid domain near the micro cylinder can be obtained. By comparing the theoretical solutions, it is found that the two-dimensional velocity distribution obtained by our simulation is similar, as Figure A2a. If different amplitudes are applied, it is found that the maximum velocity obtained by simulation is slightly larger. As the vibration amplitude increases from 0 to 24 μm, the difference between simulation solutions and theoretical solutions is more obvious, as Figure A2b.

## Appendix B. Results Supplement

### Appendix B.1. First Order Acoustic Field

In a first-order acoustic field, the oscillations of the microstructure can cause changes in temperature and pressure. The temperature change in the flow field is the result of the mutual conversion of kinetic energy and internal energy. The change in temperature is much less than one degree, which justifies the assumption that the temperature of the second-order field is constant, as Figure A3a. The pressure change is inversely symmetrical. This is due to the microstructure vibrating along the x-axis, resulting in alternating compression and expansion terms on the left and right sides, see Figure A3b.

The first-order acoustic field generated by the vibrating microstructure in the channel is influenced by the side profile. We only consider the effect on the sound pressure, and the temperature can be ignored due to its small change. From Figure A3c, as the gap increases, the maximum p1max decreases sharply and then gradually stabilizes, meantime the difference in the sound pressure distribution along the z-axis increases. It indicates that the space limited by the small gap can also amplify the sound pressure, while the amplification effect is reduced to negligible when the gap is large. The sharpening of the sidewall profile angle also can enhance the sound pressure p1max, see Figure A3d. It may be that the sharpened structure confines the fluid to the bottom of the microstructure, as shown in the inset α=80${}^{o}$.

## References

[1] Wu J. (2018). Acoustic streaming and its applications. Fluids.

[2] Luo X., Cao J., Gong H., Yan H., He L. (2018). Phase separation technology based on ultrasonic standing waves: A review. Ultrason. Sonochem..

[3] Ahmed D., Ozcelik A., Bojanala N., Nama N., Upadhyay A., Chen Y., Hanna-Rose W., Huang T.J. (2016). Rotational manipulation of single cells and organisms using acoustic waves. Nat. Commun..

[4] Collins D.J., Khoo B.L., Ma Z., Winkler A., Weser R., Schmidt H., Han J., Ai Y. (2017). Selective particle and cell capture in a continuous flow using micro-vortex acoustic streaming. Lab Chip.

[5] Ozcelik A., Nama N., Huang P.H., Kaynak M., McReynolds M.R., Hanna-Rose W., Huang T.J. (2016). Acoustofluidic rotational manipulation of cells and organisms using oscillating solid structures. Small.

[6] Ozcelik A., Rufo J., Guo F., Gu Y., Li P., Lata J., Huang T.J. (2018). Acoustic tweezers for the life sciences. Nat. Methods.

[7] Zhang H., Tang Z., Wang Z., Pan S., Han Z., Sun C., Zhang M., Duan X., Pang W. (2018). Acoustic streaming and microparticle enrichment within a microliter droplet using a Lamb-wave resonator array. Phys. Rev. Appl..

[8] Cao H.X., Jung D., Lee H.S., Go G., Nan M., Choi E., Kim C.S., Park J.O., Kang B. (2021). Micromotor Manipulation Using Ultrasonic Active Traveling Waves. Micromachines.

[9] Liu G., Lei J., Cheng F., Li K., Ji X., Huang Z., Guo Z. (2021). Ultrasonic particle manipulation in glass capillaries: A concise review. Micromachines.

[10] Lei J. (2017). Formation of inverse Chladni patterns in liquids at microscale: Roles of acoustic radiation and streaming-induced drag forces. Microfluid. Nanofluidics.

[11] Tang Q., Liu P., Guo X., Zhou S., Dong Y. (2020). 2D acoustofluidic patterns in an ultrasonic chamber modulated by phononic crystal structures. Microfluid. Nanofluidics.

[12] Tang Q., Liu P., Tang S. (2022). Rotational manipulation of massive particles in a 2D acoustofluidic chamber constituted by multiple nonlinear vibration sources. Chin. Phys. B.

[13] Takatori S.C., De Dier R., Vermant J., Brady J.F. (2016). Acoustic trapping of active matter. Nat. Commun..

[14] Endaylalu S.A., Tien W.H. (2022). A Numerical Investigation of the Mixing Performance in a Y-Junction Microchannel Induced by Acoustic Streaming. Micromachines.

[15] Huang P.H., Nama N., Mao Z., Li P., Rufo J., Chen Y., Xie Y., Wei C.H., Wang L., Huang T.J. (2014). A reliable and programmable acoustofluidic pump powered by oscillating sharp-edge structures. Lab Chip.

[16] Tang Q., Liu P., Hu J. (2018). Analyses of acoustofluidic field in ultrasonic needle-liquid-substrate system for microVnanoscale material concentration. Microfluid. Nanofluidics.

[17] Li F., Xia X., Deng Z., Lei J., Shen Y., Lin Q., Zhou W., Meng L., Wu J., Cai F. (2019). Ultrafast Rayleigh-like streaming in a sub-wavelength slit between two phononic crystal plates. J. Appl. Phys..

[18] Wiklund M., Green R., Ohlin M. (2012). Acoustofluidics 14: Applications of acoustic streaming in microfluidic devices. Lab Chip.

[19] Laubli N.F., Gerlt M.S., Wuthrich A., Lewis R.T.M., Shamsudhin N., Kutay U., Ahmed D., Dual J., Nelson B.J. (2021). Embedded microbubbles for acoustic manipulation of single cells and microfluidic applications. Anal. Chem..

[20] Ahmed D., Mao X., Juluri B.K., Huang T.J. (2009). A fast microfluidic mixer based on acoustically driven sidewall-trapped microbubbles. Microfluid. Nanofluidics.

[21] Volk A., Rossi M., Kähler C.J., Hilgenfeldt S., Marin A. (2015). Growth control of sessile microbubbles in PDMS devices. Lab Chip.

[22] Belling J.N., Heidenreich L.K., Tian Z., Mendoza A.M., Chiou T.T., Gong Y., Chen N.Y., Young T.D., Wattanatorn N., Park J.H. (2020). Acoustofluidic sonoporation for gene delivery to human hematopoietic stem and progenitor cells. Proc. Natl. Acad. Sci. USA.

[23] Rodamporn S., Harris N., Beeby S.P., Boltryk R.J., Sanchez-Eisner T. (2010). HeLa cell transfection using a novel sonoporation system. IEEE Trans. Biomed. Eng..

[24] Hayakawa T., Sakuma S., Arai F. (2015). On-chip 3D rotation of oocyte based on a vibration-induced local whirling flow. Microsystems Nanoeng..

[25] Hayakawa T., Sakuma S., Fukuhara T., Yokoyama Y., Arai F. (2014). A single cell extraction chip using vibration-induced whirling flow and a thermo-responsive gel pattern. Micromachines.

[26] Feng L., Song B., Zhang D., Jiang Y., Arai F. (2018). On-chip tunable cell rotation using acoustically oscillating asymmetrical microstructures. Micromachines.

[27] Huang P.H., Xie Y., Ahmed D., Rufo J., Nama N., Chen Y., Chan C.Y., Huang T.J. (2013). An acoustofluidic micromixer based on oscillating sidewall sharp-edges. Lab Chip.

[28] Zhao S.K., Hu X.J., Zhu J.M., Luo Z.Y., Liang L., Yang D.Y., Chen Y.L., Chen L.F., Zheng Y.J., Hu Q.H. (2021). On-chip rapid drug screening of leukemia cells by acoustic streaming. Lab Chip.

[29] Lu X., Martin A., Soto F., Angsantikul P., Li J., Chen C., Liang Y., Hu J., Zhang L., Wang J. (2019). Parallel label-free isolation of cancer cells using arrays of acoustic microstreaming traps. Adv. Mater. Technol..

[30] Shen H., Zhao K., Wang Z., Xu X., Lu J., Liu W., Lu X. (2019). Local Acoustic Fields Powered Assembly of Microparticles and Applications. Micromachines.

[31] Wang G., Yang F., Zhao W. (2016). Microelectrokinetic turbulence in microfluidics at low Reynolds number. Phys. Rev. E.

[32] Lu X., Zhao K., Peng H., Li H., Liu W. (2019). Local enhanced microstreaming for controllable high-speed acoustic rotary microsystems. Phys. Rev. Appl..

[33] Lieu V.H., House T.A., Schwartz D.T. (2012). Hydrodynamic tweezers: Impact of design geometry on flow and microparticle trapping. Anal. Chem..

[34] Zhang C., Guo X., Brunet P., Costalonga M., Royon L. (2019). Acoustic streaming near a sharp structure and its mixing performance characterization. Microfluid. Nanofluidics.

[35] Doinikov A.A., Gerlt M.S., Pavlic A., Dual J. (2020). Acoustic streaming produced by sharp-edge structures in microfluidic devices. Microfluid. Nanofluidics.

[36] Mohanty S., Siciliani de Cumis U., Solsona M., Misra S. (2019). Bi-directional transportation of micro-agents induced by symmetry-broken acoustic streaming. AIP Adv..

[37] Zhang C., Guo X., Royon L., Brunet P. (2020). Unveiling of the mechanisms of acoustic streaming induced by sharp edges. Phys. Rev. E.

[38] Lei J., Hill M., de León Albarrán C.P., Glynne-Jones P. (2018). Effects of micron scale surface profiles on acoustic streaming. Microfluid. Nanofluidics.

[39] Kaneko K., Osawa T., Kametani Y., Hayakawa T., Hasegawa Y., Suzuki H. (2018). Numerical and experimental analyses of three-dimensional unsteady flow around a micro-pillar subjected to rotational vibration. Micromachines.

[40] Yang R., Wang W. (2005). A numerical and experimental study on gap compensation and wavelength selection in UV-lithography of ultra-high aspect ratio SU-8 microstructures. Sens. Actuators B Chem..

[41] Lei J., Glynne-Jones P., Hill M. (2017). Comparing methods for the modelling of boundary-driven streaming in acoustofluidic devices. Microfluid. Nanofluidics.

[42] Holmes M., Parker N., Povey M. (2011). Temperature dependence of bulk viscosity in water using acoustic spectroscopy. J. Phys. Conf. Ser..

[43] Muller P.B., Barnkob R., Jensen M.J.H., Bruus H. (2012). A numerical study of microparticle acoustophoresis driven by acoustic radiation forces and streaming-induced drag forces. Lab Chip.

[44] Vernekar V.N., Cullen D.K., Fogleman N., Choi Y., García A.J., Allen M.G., Brewer G.J., LaPlaca M.C. (2009). SU-8 2000 rendered cytocompatible for neuronal bioMEMS applications. J. Biomed. Mater. Res. Part A Off. J. Soc. Biomater. Jpn. Soc. Biomater. Aust. Soc. Biomater. Korean Soc. Biomater..

[45] Ribeiro J., Minas G., Turmezei P., Wolffenbuttel R., Correia J. (2005). A SU-8 fluidic microsystem for biological fluids analysis. Sens. Actuators A Phys..

[46] Nemani K.V., Moodie K.L., Brennick J.B., Su A., Gimi B. (2013). In vitro and in vivo evaluation of SU-8 biocompatibility. Mater. Sci. Eng. C.

[47] Anderson J.D. (1992). Governing equations of fluid dynamics. Computational Fluid Dynamics.

[48] Liu C. (2021). New ideas on governing equations of fluid dynamics. J. Hydrodyn..

[49] Muller P.B. (2012). Acoustofluidics in Microsystems: Investigation of Acoustic Streaming. Master’s Thesis.

[50] Karlsen J.T., Bruus H. (2015). Forces acting on a small particle in an acoustical field in a thermoviscous fluid. Phys. Rev. E.

[51] Hintermüller M.A., Reichel E.K., Jakoby B. (2017). The influence of a background flow on acoustic streaming. Proceedings of the 2017 IEEE International Ultrasonics Symposium (IUS).

[52] Bruus H., Laurell T., Lenshof A. (2014). Perturbation theory and ultrasound resonances. Microscale Acoustofluidics.

[53] Hayakawa T., Akita Y., Arai F. (2018). Parallel trapping of single motile cells based on vibration-induced flow. Microfluid. Nanofluidics.

[54] Zhang C., Guo X., Royon L., Brunet P. (2020). Acoustic streaming generated by sharp edges: The coupled influences of liquid viscosity and acoustic frequency. Micromachines.

